# Maternal Dietary Protein Intake Influences Milk and Offspring Gut Microbial Diversity in a Rat (*Rattus norvegicus*) Model

**DOI:** 10.3390/nu11092257

**Published:** 2019-09-19

**Authors:** Matthew F. Warren, Haley A. Hallowell, Keah V. Higgins, Mark R. Liles, Wendy R. Hood

**Affiliations:** 1 Department of Biological Sciences, Auburn University, Auburn, AL 36849, USA; mfwarren@ncsu.edu (M.F.W.); hah0024@tigermail.auburn.edu (H.A.H.); kvc0004@tigermail.auburn.edu (K.V.H.); lilesma@auburn.edu (M.R.L.); 2 Prestage Department of Poultry Science, North Carolina State University, Raleigh, NC 27695, USA

**Keywords:** Milk microorganisms, bacteria diversity, phylogenetic distance, 16S rRNA gene sequencing

## Abstract

Historically, investigators have assumed microorganisms identified in mother’s milk to be contaminants, but recent data suggest that milk microbiota may contribute to beneficial maternal effects. Microorganisms that colonize the gastrointestinal tracts of newborn mammals are derived, at least in part, from the maternal microbial population. Milk-derived microbiota is an important source of this microbial inocula and we hypothesized that the maternal diet contributes to variation in this microbial community. To evaluate the relationship between a mother’s diet and milk microbiome, we fed female rats a low- or high-protein diet and mated all individuals. Milk and cecal contents were collected from dams at peak lactation (14-day post-partum), and the bacterial composition of each community was assessed by 16S rRNA gene amplicon sequencing. Our findings revealed higher dietary protein intake decreased fecal microbial diversity but increased milk microbial and pup cecum diversity. Further, the higher dietary protein intake resulted in a greater abundance of potentially health-promoting bacteria, such as *Lactobacillus* spp. These data suggest that dietary protein levels contribute to significant shifts in the composition of maternal milk microbiota and that the functional consequences of these changes in microbial inocula might be biologically important and should be further explored.

## 1. Introduction

Nutrients, antibodies, and other bioactive compounds that are transferred from mother to offspring in milk are instrumental to offspring development and future performance [1,2]. While there is a long history of investigating milk microbiota to avoid pathogens, such as *Listeria monocytogenes*, it is now better appreciated that milk-derived bacteria contribute to a diverse microbial community that has important functional roles in the gastrointestinal tract of offspring [3,4]. Enteric microorganisms contribute to variation in digestive efficiency and immune function of developing young [1], making it important to understand what factors contribute to variation of the microbes found within the milk microbiome.

A number of factors contribute to variation in the composition of bacteria within the milk microbiome, including a mother’s environment, her mode of delivery at parturition, and her body composition [2,3,5]. Microorganisms entering the mammary gland are derived from populations residing on the mother’s skin surrounding the nipple, mouth of offspring, and environment. Perez et al. [6] suggested that microorganisms in milk might also be transported from the mother’s gastrointestinal tract to the milk in her mammary glands via an entero-mammary pathway that includes mucosal dendritic cells associated with the mother’s immune system [7]. This could implicate microbial transfer from gut to mammary gland as an additional maternal effect on offspring nutrition. Alternatively, the maternal diet may influence nutrients available to microorganisms and immune system functionality in the mammary gland, thereby affecting the composition of the milk microbiome. Prior to testing specific hypotheses on maternal influences on milk microbiome, it is important to evaluate the influence of maternal diet on the microbial composition of milk.

Given potential sources of microorganisms in a mother’s milk, several factors could contribute to interactions between a mother’s diet and her milk-associated microbiota. In searching for and obtaining food, women and animals may be introduced to different microorganisms in their environment. The quality of a mother’s diet can contribute to variation in composition and volume of milk that she produces [8], and this source of nutrients may influence what microorganisms can survive and thrive within the mammary gland. Diet can also have a strong impact on intestinal microbial diversity [9,10]. In Perez et al.’s [6] study, they cultured bacteria from feces, blood, and milk of women and found that *Bifidobacterium longus*, *Streptococcus thermophiles*/*salivarius*, and *Staphylococcus epidermidis* were persistent across all three sample types. Despite the fact that the methods used in this study were not the current state-of-the-art methods used in microbiome research and the three species evaluated represented a small fraction of those microbial species found within mothers’ milk, the conclusions of this study have been presented as dogma in a number of recent reviews [5,11,12]. Before more detailed mechanistic studies are performed to explore potential bacterial transport from gut to mammary gland, it is important to assess the effects of diet on enteric and mammary-associated microorganisms. To evaluate this relationship, we compared maternal milk and both maternal and pup enteric microbiota of rats fed diets that differed in relative protein content.

To examine the relative effects of protein intake on milk and enteric bacterial diversity, we first conducted a pilot study with female rats to compare the impact of 10%, 15%, and 20% protein isocaloric diets on gut bacteria by quantifying fecal bacterial diversity with 16S rRNA gene sequencing. Carbohydrates (corn starch) were used to make up the difference in caloric content of two diets. Thus, the 10% protein diet was not only low in protein, but it was higher in carbohydrates. Preliminary results showed few significant differences in fecal bacterial diversity for rats consuming 10% or 15% protein diets, but greater variation was observed between rats consuming 10% protein diet or a 20% protein diet (Appendix A). For the main study, we chose to evaluate the impact of protein intake on milk microbial diversity for rats fed 10% and 20% protein diets. Rats consuming low-protein (LP) were predicted to have significantly low bacterial diversity than rats consuming the high-protein diet (HP). Particular attention was paid to *Bifidobacterium*, *Streptococcus*, and *Staphylococcus* that have been previously proposed to be transferred via the hypothesized entero-mammary pathway. Support for this hypothesis would come from experimental data indicating shared operational taxonomic units (OTUs) that would simultaneously occur in milk and the intestinal tract of lactating females and their pups.

## 2. Materials and Methods

Auburn University’s Institutional Animal Care and Use Committee, protocol number 2014-2544, approved all methods for this study.

### 2.1. Animal and Housing Conditions and Diet

Sprague–Dawley rats (*Rattus norvegicus*) were selected for this study because of their docility, large litters, and strong maternal instinct [9,10]. Rat dams also produce copious amount of milk relative to smaller rodents. Ten-week-old Sprague–Dawley rats obtained from Envigo Laboratories (Indianapolis, IN, USA) were used for both the preliminary and the primary experiment. Rats were housed at Auburn University’s Biological Research Facility under specific pathogen-free and standard housing conditions (14:10 light cycle, 21 °C). Only females were used in the preliminary experiment, and all females were housed in pairs. For the primary experiment, females were housed with males until late pregnancy, when the male was removed. Data collection was limited to females in all experiments.

### 2.2. Diets

Three different custom rodent diets that varied in relative protein content, low-protein (LP), moderate-protein (MP), and high-protein (HP), were obtained from TestDiet (Purina Animal Nutrition, LLC., St. Louis, MO, USA). All three diets were plant-based to feed the rats a diet that had greater similarity to the natural diet of wild *Rattus norvegicus* than the milk-protein rich diets commonly fed to laboratory rodents. Food and water were offered to rats *ad libitum* in both experiments. Diets were formulated with ground corn, soybean meal (absent from LP), ground wheat, fish mean (absent LP), wheat middlings, dried beet pulp, cane molasses, wheat germ, brewers dried yeast, dehydrated alfalfa meal, ground oats, soybean oil, and whey. Vitamins and minerals were also consistently added to all diets following standards for laboratory rodent. Variation in the relative amount of fish meal and soybean contributed to differences in the protein content of diets. Diets were made isocaloric by adjusting the amount of wheat and soybean in each diet. Final diets differed in the relative amount of starch but were comparable in percent neutral detergent fiber, acid detergent fiber, and both mono- and disaccharides (Table 1). The results of the preliminary diet trial are presented in the supplemental material. Because the fecal bacterial community for rats consuming the moderate-protein diet was not different from the other two groups (Appendix A), we selected the LP and HP diet for the main study.

### 2.3. Milk and Cecal Microbiota Experiment

Sixteen seven-week-old female rats were randomly assigned to one of the two treatment groups, LP and HP (*n* = 8 female rats/diet; *n* = 4 male rats/diet were used for mating). Females were supplied with a cardboard bio-home (Bio-Serv, Flemington, NJ, USA) to encourage nest building. Two weeks after pairing males and female rats, the females were monitored for increasing girth associated with pregnancy and moved to an isolated cage in late pregnancy. Females were then checked daily for the presence of newborn pups. The day of birth for each female was described as 1-day post-partum.

Milk was collected from each dam at peak lactation (14-days post-partum) [8]. At the time of collection, each dam was separated from her litter and moved to a different cage with *ad lib* access with food and water, allowing milk to accumulate. When 3 hours passed, the dam was injected with 5 IU/mL of oxytocin (Osborn, Bimeda Inc, Oakbrook Terrace, IL, USA, concentration = 20 USP per mL) intramuscularly to stimulate milk letdown. The dam was then placed with her litter for 2 to 3 minutes, allowing the pups to suckle and enhance milk letdown. Dams were anesthetized by placing them in a 4 L glass jar containing cotton balls presoaked with isoflurane. Each dam’s teats were cleaned with ethyl alcohol, and milk was gently expressed from 6 teats from the right side by manual palpation into a sterile capillary tube. Milk was transferred with a capillary tube to a screw-top microcentrifuge tube and was flash-frozen in liquid nitrogen. Rats were killed by decapitation, and cecal contents were collected aseptically from dams and one random male and female pup from each litter. Prior research in mice has suggested that the microbial composition of the small and large intestine, cecum, and feces are similar [13]. All samples were stored in an −80 °C freezer for future analysis. Two females were removed from the study before milk was collected, one never became pregnant, and the second cannibalized her litter.

### 2.4. Common Laboratory and Bioinformatics Methods

Milk samples were thawed, vortexed, and subsampled for 16S rRNA gene sequencing using a milk microbial DNA extraction kit (Norgen Biotek Corp, Thorold, ON, Canada) with 200 µL milk. Cecal DNA extractions were completed using the E.Z.N.A. Stool DNA Kit (Omega Biotek, Norcross, GA, USA) with approximately 0.2 g of fecal or cecal material. Because the amount of cecal material collected from the pups was often <0.2 g, a randomly selected male and female pup from each litter were selected and pooled within the litter, with approximately equal amounts of sample contributing the pool from each pup. Extracted genomic DNA (gDNA) samples were quantified using a Nanodrop (Thermo Scientific, Wilmington, DE, USA) to verify DNA concentration, and 40 µL replicates were sent to Molecular Research LP (Shallowater, TX, USA) for 16S rRNA gene amplicon sequencing (targeting variable region 4) using an Illumina MiSeq (San Diego, CA, USA) next-generation sequencer.

Raw 16S rRNA gene amplicon sequences were trimmed for quality and analyzed in the pipeline available through using Quantitative Insights Into Microbial Ecology (QIIME) version 1.9.0 and VSEARCH version 1.4 [14] to create files for downstream bacterial relative abundance and diversity analyses [15,16]. Paired-end sequences were joined and quality filtered to contain sequences between 300–570 base pairs, and all sequences outside that range were discarded, and the mapping file was formatted. Barcodes were extracted, and sequences were demultiplexed for downstream analyses. Reads were dereplicated, and an OTU table was generated for chimera detection. Chimeras were detected through de novo checking and then used to filter out chimeras in the OTU table. Taxonomy was assigned to OTUs using the Greengenes reference database with a 97% threshold of pairwise identity. Alpha (diversity within samples) and beta diversity (diversity between samples), non-metric multidimensional scaling (nMDS), and relative abundances of taxa were analyzed using diversity analysis in QIIME. nMDS was used to compare groups of samples using count-based distance matrices to the rank distance between samples and generate axes that optimize differences between samples to visualize how samples are placed to explore relationships between samples [17].

### 2.5. Statistical Analyses

Statistical analyses were done using R version 3.5.2 [18] and SAS software, Version 9.4 (Cary, NC, USA). General linear models (GLM) were used to compare sample type and dietary group in a rarefaction plot, and GLM was used to analyze the alpha diversity of bacterial communities between the same groups. Alpha diversity parameters measured were Shannon–Wiener Index, phylogenetic distance, and unique OTUs. Beta diversity was evaluated between same groups with nMDS using Bray–Curtis dissimilarity as a parameterizing index. GLM was also used to test for variation between groups of each OTU. An interaction term was also included in each of these GLM models. Statistical significance was established at *p* < 0.05.

## 3. Results

### 3.1. Alpha and Beta Diversity for Milk, and Dam and Offspring Cecal Contents

**Figure 1:**
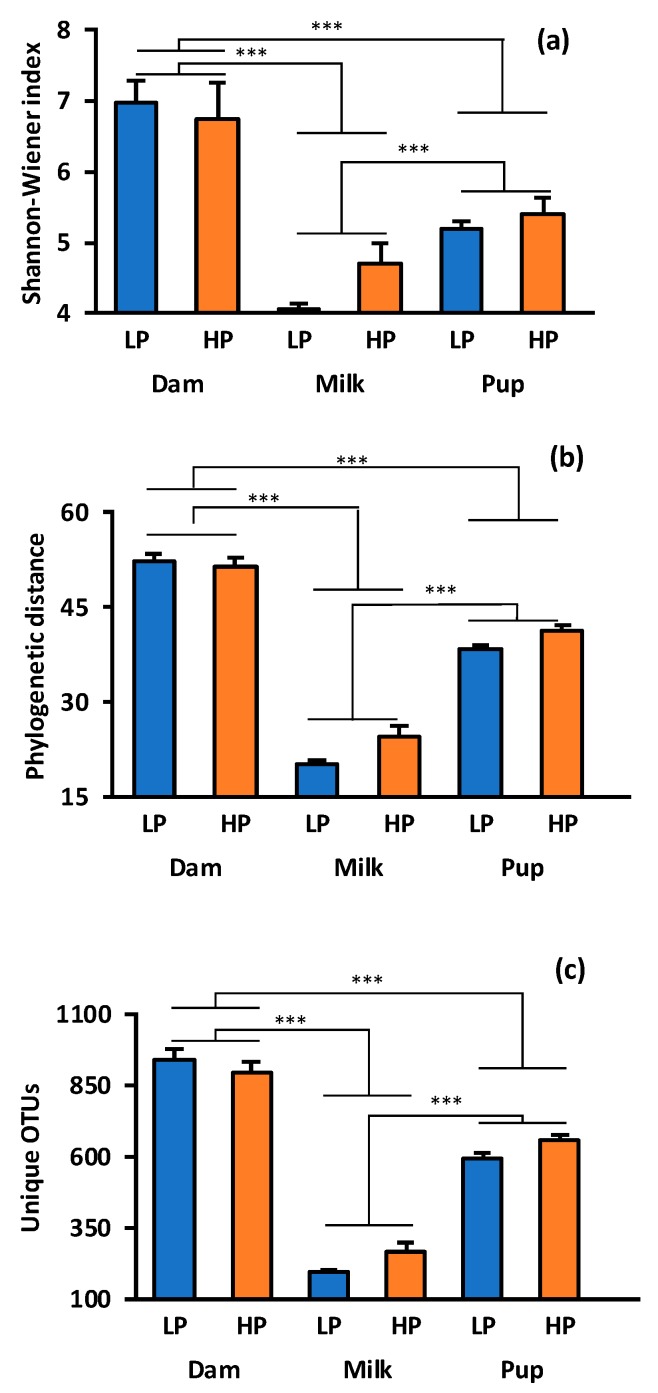
Comparison of bacterial OTUs (operational taxonomic units) for the milk and cecum of rats, consuming a 10% protein (low-protein (LP), blue bars) vs. 20% protein (high-protein diet (HP), orange bars) diet, and their pups. (**a**) Alpha diversity by location and diet using the Shannon–Wiener Index. (**b**) Alpha diversity by sample type and diet using phylogenetic distance. (**c**) Alpha diversity by location and diet using unique OTUs. Bar graphs show means and standard error bars. An asterisk indicates statistical difference (General linear models (GLM), *** *p* ≤ 0.0001).

A total of 4,935,494 reads were produced by 16S rRNA gene amplicon sequencing after quality-filtering and a mean sample depth of 88,133 reads per sample with a standard deviation of 34,066 reads from the milk and cecal samples. A total of 17,616 randomly selected sequences from all samples were used to evaluate alpha and beta diversity. A rarefaction plot was generated to determine if sampling depth was adequate to capture most, if not all, OTUs in these samples (Figure S1). Relative diversity of bacterial assemblages was different between the three samples types that were collected, including the maternal cecum, maternal milk, and offspring cecum. Asymptotes were observed for each of these sample types based on rarefaction curves, indicating that the degree of sampling was sufficient to account for most OTUs present in these bacterial assemblages. Comparable to rarefaction analysis, variation in alpha diversity was largely driven by the sample type. The Shannon–Wiener diversity index (overall: F = 15.3, degrees of freedom (df) = 5, 50, *p* < 0.0001, Figure 1a) was highest in maternal cecal samples, intermediate in pup cecal samples, and lowest in milk (sample type: F = 36.8, df = 2, 55, *p* < 0.0001), but Shannon–Wiener index did not vary with relative protein content of the diet (*p* = 0.35). Phylogenetic distance varied with sample type collected (overall: F = 124, df = 5, 50, *p* < 0.0001; sample type: F = 303, df = 2, *p* < 0.0001, Figure 1b), and diet with HP samples displayed greater distance than LP samples (F = 5.33, df = 1, *p* = 0.025). Unique OTUs followed the same pattern, differing with sample type collected (overall: F = 126, df = 5,50, *p* < 0.0001; sample type: F = 309, df = 2, *p* < 0.0001, Figure 1c) and not dietary treatment (diet: *F* = 3.79, df = 1, *p* = 0.057).

**Figure 2:**
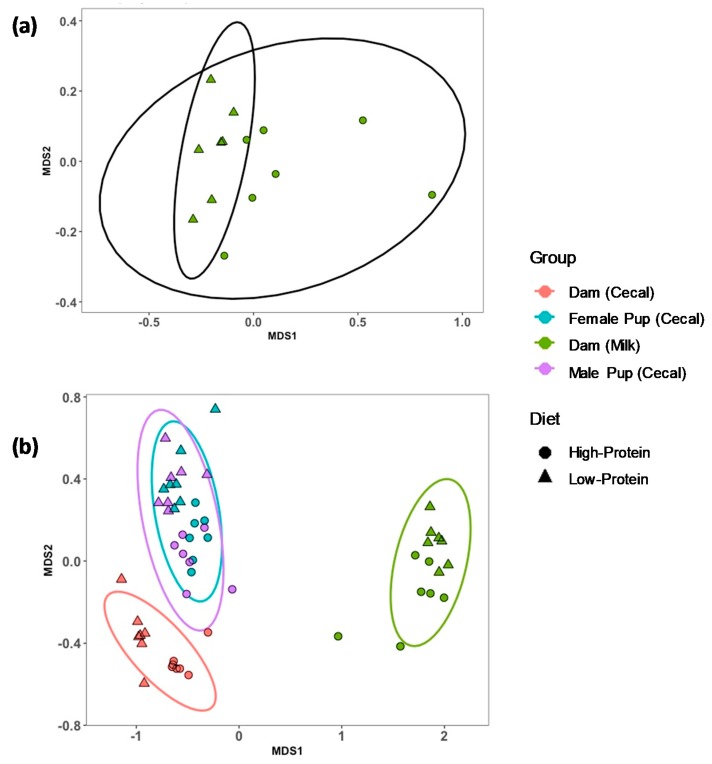
Non-metric multidimensional scaling (nMDS) plots of (**a**) rat dam milk samples grouped by diet and (**b**) rat dam cecum, pup cecum, and dam milk. Ten percent protein (LP) diet samples denoted by triangles and 20% protein (HP) diet samples denoted by circles. Bacterial OTUs were clustered using Bray–Curtis. Stress was (**a**) 0.12 and (**b**) 0.07.

For measures of beta diversity, the results of Bray–Curtis ANOSIM (analysis of similarities) evaluated combined relationships between sample type collected and dietary treatment, and they were significant (*r* = 0.84; *p* = 0.001). LP milk samples clustered with separation from HP milk samples, signifying dietary impacts on bacterial populations in milk with the stress of 0.12 (Figure 2a). An nMDS plot with dam cecal, milk, and pup cecal samples together had the stress of 0.07, and distinct clusters with community location were formed along MDS1 (Figure 2b). Dam cecal samples exhibited little variation within dietary treatment groups as HP samples were tightly clustered and vice versa for LP samples (Figure S2a). Pup cecal samples exhibited mirroring between HP and LP samples, signifying dietary impact on bacteria in their gut (Figure S2b). Within each of these clusters, the distance between the sample types collected was much greater than the distance between dietary groups, suggesting the sample type has a greater role in microbial composition than a maternal diet. 

### 3.2. Impact of Maternal Diet on Specific Microorganisms between Maternal Ceca, Milk, and Offspring Ceca

As predicted, the sample type had a significant impact on bacterial phyla and OTU relative abundance (Table 2 and Table 3). The relative abundance of several OTUs in milk were significantly different between dietary treatment groups. Milk bacterial assemblages were dominated by the phyla Actinobacteria, Firmicutes, and Proteobacteria, whereas the phyla Bacteroidetes and Verrucomicrobia were absent (Table 2 and Table 3). For both maternal and offspring cecal samples, Bacteroidetes and Firmicutes were most abundant, Actinobacteria were negligible, and Verrucomicrobia were more abundant in ceca offspring compared to ceca of their mothers (Table 2 and Table 3). Firmicutes and Verrucomicrobia were both affected by dietary treatment. As a whole, members of the phyla Bacteroidetes and Proteobacteria were not significantly impacted by diet (Table 2 and Table 3).

**Figure 3:**
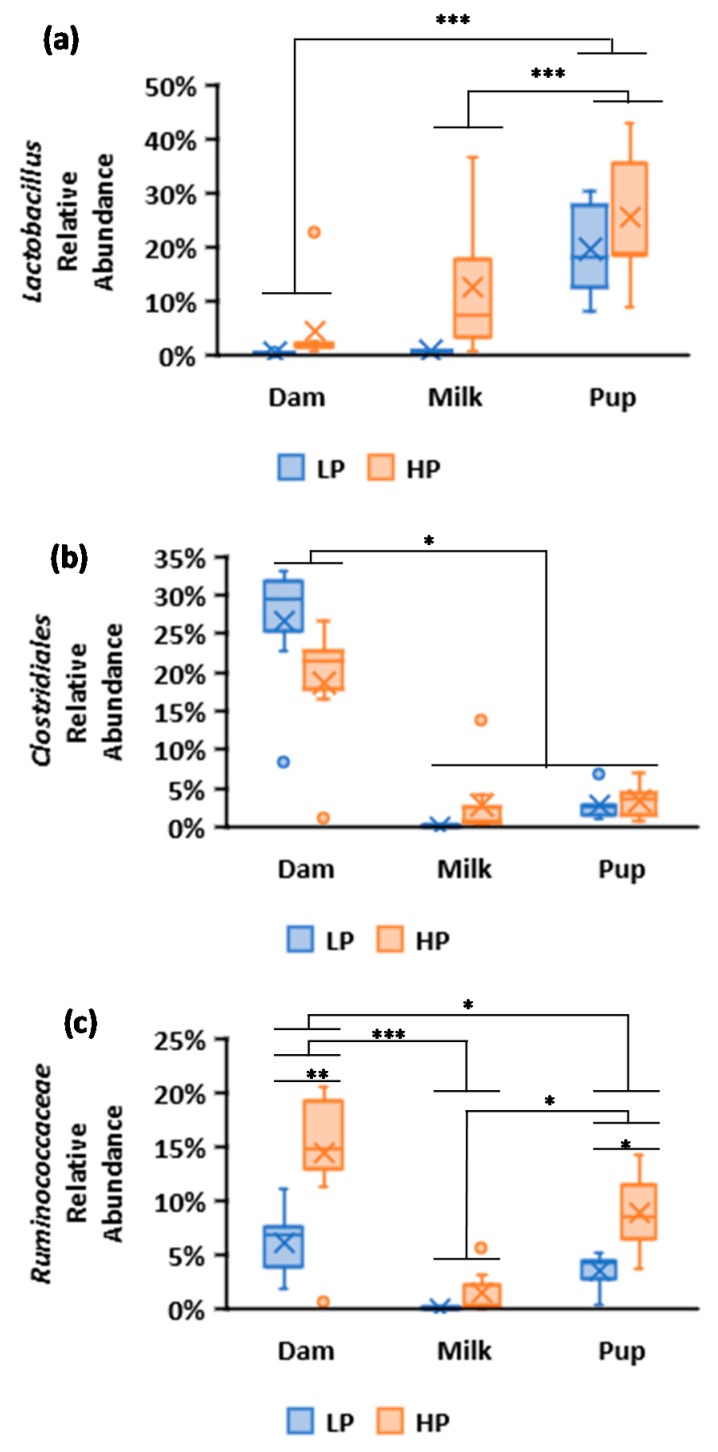
Box-and-whisker plots of relative abundances of OTUs affected by maternal dietary protein intake from rat dam cecum, milk, and pup cecum. Blue boxes denote 10% protein (LP), and orange boxes denote 20% protein (HP) with X’s denoting means. (**a**) *Lactobacillus*, (**b**) *Clostridiales*, (**c**) *Ruminococcaceae* (GLM, * *p* < 0.05; ** *p* ≤ 0.001; *** *p* ≤ 0.0001).

Only five OTUs were affected by dietary treatment group. Of these, *Ruminococcus* was negligible, and Verrucomicrobia were absent in milk; *Haemophilus* and *Mannheimia* were negligible in cecal samples (Table 2 and Table 3). The remaining two OTUs affected by diet were *Lactobacillus* and *Ruminococcaceae* (Table 3). *Lactobacillus* spp. were consistently observed in HP groups. In pups, *Lactobacillus* spp. made up more than at least 20% of OTUs in offspring for both diets, but were insignificant in cecum and milk of LP-fed dams (Figure 3, Table 2). *Clostridiales* taxa were observed to have significant interaction between sample type and diet, which reflects higher relative abundance in the cecum of LP dams than HP dams, but conversely were observed to have a higher relative abundance of *Clostridiales* in the milk of HP dams compared to LP dams. *Clostidiales* in offspring ceca were not different between dietary treatments.

## 4. Discussion

The results of this investigation suggested that a mother’s diet impacts thebacterial taxa found in milk and that the maternal diet is associated with differences in the bacterial community in the dam’s cecum and the cecum of their offspring. Consistent with other studies [19,20,21,22], the milk-associated bacteria were observed to have a significantly lower number of unique OTUs and had lower alpha and beta diversity compared to that of bacterial taxa in the maternal and pup ceca. Pups had decreased gut bacterial diversity compared to dams, as anticipated because microbial colonization of the gut takes time to establish and specific microorganisms either dominate the gut or are removed as offspring age [23,24,25,26]. The bacteria found in rat milk were almost equally represented by the phyla Actinobacteria, Firmicutes, and Proteobacteria, with Actinobacteria taxa being slightly less abundant than the other two groups.

The most abundant OTUs found in rat milk were *Rothia*, which accounted for most of the hits within phylum Actinobacteria. As many *Rothia* spp. are opportunistic pathogens, the high relative abundance of these bacteria in milk are of potential concern, especially if animals encounter stressful conditions that lead to an immunocompromised state [27]. Given that *Rothia* spp. are commonly found in the oral cavity and skin, it is possible that the *Rothia* taxa in these samples originated from dam teats or offspring mouths [28,29]. *Rothia* spp. was only observed to have a relative abundance of <0.5% in the ceca of mothers and was only slightly higher than that in the ceca of pups.

*Agrobacterium* spp. accounted for most of Proteobacteria taxa observed, and this genus includes, but is not limited to, common plant pathogens that cause crown-gall tumors in plants [30]; it is unknown if the observed OTU is a plant pathogen or benign. McInnis et al. [31] reported the presence of *Agrobacterium* and other Proteobacteria in raw goat milk, but their relevance to health and milk quality are unknown. A few *Agrobacterium* spp. have been found in termite guts [30], and the small intestines of Arctic char fed high levels of carbohydrates. In these studies, *Agrobacterium* was speculated to contribute to nutritional processes [32], yet *Agrobacterium* was not found in the cecum of dams or their pups. We believe *Agrobacterium* found in rat milk is associated with uptake from their plant-based diet or pine contact bedding in their boxes. This could be confirmed by evaluating the bacteria associated with the rat’s diets and bedding.

Among the bacterial taxa from milk observed within the phylum Firmicutes, the genera *Staphylococcus, Lactobacillus*, and *Streptococcus* were dominant. Only two OTUs found at concentrations greater than 1% were similarly influenced by diet within the cecum of dams’, their milk, and the cecum of their offspring, which were affiliated with the genus *Lactobacillus*, and family *Ruminococcaceae*. While in the study by Perez et al., members of the genera *Staphylococcus, Streptococcus*, and *Bifidobacterium* were identified as potentially transported via an entero-mammary pathway [6], in this study, the only abundant taxa that were significantly affected by diet and found in ceca of dams and their milk were *Lactobacillus* and *Ruminococcaceae* taxa. Both *Lactobacillus* and *Ruminococccaceae* were higher in ceca and milk of mothers consuming an HP diet. While we cannot rule out an entero-mammary transmission of these OTUs, proximity alone could easily be responsible for this relationship. *Clostridiales*, *Ruminococcaceae*, and *Lactobacillus* are common gut microbial taxa that inhabit the gut microbiome. *Lactobacillus* spp. are common in fecal and milk microbiota across species and also produce bacteriocins to inhibit the growth of pathogens in milk [33]. *Ruminococcaceae* comprises obligate anaerobes in colons that are associated with colonizing mucosal folds, and many *Ruminococcus* spp. inhabiting mammalian guts are known to be capable of degrading cellulose and other complex polysaccharides associated with a plant-based diet [34,35]. *Clostridiales* spp. were observed to have a greater relative abundance in dams that consumed the LP diet, while conversely, their relative abundance was greater in pups that consumed the HP diet. *Clostridiales* are spore-forming, obligate anaerobes and include both pathogenic (*C. difficile*) and commensal (*C. butyricum*) species, with significant variability, observed even at the strain level among *Clostridium* spp. [36]. Many species of *Clostridiales* are capable of producing potent toxins [37,38]. Without further phylogenetic resolution or detection of toxin-producing genes associated with these *Clostridiales* taxa, it is not possible to attribute any functional significance to these changes in their relative abundance.

A potential reason why HP dam ceca had reduced abundance of *Clostridiales* is that the relationship is a product of changes in the luminal environment under high dietary protein intake. Liu et al. [39] similarly found reduced abundance of *Clostridia* in the lumens of ceca and colons of rats fed a 53% versus a 14% protein diets. Further, Zhou et al. [40] observed that the ceca of pigs consuming low dietary protein had higher relative abundances of unclassified *Clostridiaceae* relative to pigs that consumed a standard protein diet. It has been suggested that the production of short-chain fatty acids (SCFA) by microbes such as *Bacteroides* could impact microbial community dynamics [39,40]. While Zhou et al. [40] found lower production of SCFA in ceca of pigs fed the lower protein diets, it was not clear if SCFAs affected the relative abundance of *Clostridiaceae*. In our study, the relative abundance of *Clostridiales* was also much lower in the milk and pup cecum, but it was not statistically different between diets. Two studies that also examined milk microbiota reported low relative abundance with *Clostridium* spp. [41,42]. Further, the relative abundance of clostridia in bacterial communities fed synbiotic diets has been shown to decrease when lactobacilli were included in the diet, suggesting a possible antagonistic relationship [43].

*Lactobacillus* is common in the fecal and milk microbiome across species and also produce bacteriocins to inhibit the growth of pathogens in milk [32]. Multiple species of *Staphylococcus* have also been identified in milk, including commensal and pathogenic strains [28]. Unfortunately, the limited phylogenetic resolution of 16S rRNA gene sequences, prevented us from identifying the species of *Staphylococcus* found in rat milk. *Staphylococcus* was more abundant in the milk microbial communities of dams consuming an LP diet, and *Lactobacillus* spp. were observed to be more abundant in milk from animals consuming an HP diet. *Staphylococcus aureus* is known to compete with lactic acid bacteria like *Lactobacillus* for nutrients, and *S. aureus* can metabolize different carbon sources, including lactose, to facilitate its growth in milk [44]. It is possible this interaction contributed to differences in relative abundances between diets for these two bacteria.

The results of the nMDS suggest that the bacterial assemblages found in dams’ milk, her cecum, and the ceca of her pups are distinct. Even though no dietary effect was observed for alpha diversity, beta diversity was significant for sample type and diet, with the bacteria found in different sample types being more disparate than communities collected from the same sample type from animals consuming different diets. These results reinforce prior findings, which suggest that the microenvironment of each microbial community is often more important than environmental variation in determining microbial composition [45,46,47]. Any similarities between bacterial assemblages and treatment group could be determined by the proximity of each to the food and feces of the consumers.

There remain many unresolved questions concerning the factors that contribute to microbial composition and persistence within mammary tissues. Due to immune surveillance, mammary tissue is generally an unsuitable environment for bacterial replication and persistence [48]. Maternal immune cells travel into mammary tissue and are present in milk [49], and thus, any microbes that make it into the milk alveoli are likely to be destroyed. In this study, milk was collected from the same route as suckling offspring. While cleaning the teat with alcohol before milk collection would have reduced some of the bacteria accumulating on the skin, the samples we collected would be replete with microbes residing in the galactophores that milk passes through in route from the milk alveoli to the nipple. With the barriers to microbial colonization and persistence within mammary tissues in mind, the concept of an “entero-mammary pathway” resulting in the presence of enteric microbes within the mammary ducts is problematic, especially due to dendritic cell processing of bacterial cells and their migration to lymph nodes to elicit an immune response [50,51]. A recent study examined if pregnant women taking a probiotic would have altered milk microbiota and reported no difference in lactobacilli or bifidobacterial abundance in breast milk between women taking placebo or probiotic [52]. Mastromarino et al. [52] suggested that oral intake of probiotic microbes did not pass from maternal gut to mammary gland, but could alter microbiota depending on the mode of delivery.

While we have not directly evaluated the postulated entero-mammary pathway in this study, our findings suggested that it would be limited biological importance at peak lactation, if present at all. We speculate that milk microbiota is largely derived from exogenous sources. This is supported by a recent study by Moossavi et al. [53] who suggested that the offspring microbiota, particularly from the oral cavity, altered the bacteria expressed in milk. Further, any impact that diet has on the composition of milk is likely to impact the abundance of bacterial species. Indeed, it is well known that relative maternal protein intake can alter the protein content of milk [54]. We observed a difference in milk-associated bacteria with differences in maternal protein intake. Future research should directly evaluate how variation in milk composition impacts milk microbial community.

## 5. Conclusions

Our work highlights how dietary protein intake can shift bacterial diversity in rat feces, ceca, and milk and potentially select for beneficial microorganisms to be passed onto offspring. High-protein intake appeared to have a greater abundance of potentially beneficial bacteria found in ceca and milk, such as *Lactobacillus* spp. Future work should focus on evaluating the mechanisms by which diet impacts mammary-associated microbiota.

## Figures and Tables

**Table 1:** Macronutrient profile of the custom diets used in this experiment (TestDiet, Purina Animal Nutrition, LLC., St. Louis, MO, USA), including low-protein, moderate-protein, and high-protein.

Components ^#^	Low-Protein	Moderate-Protein	High-Protein
Dry matter (DM, % as is)	90.5	90.6	90.5
Crude protein (% DM)	10.3	15.1	20.9
Fat (acid hydrolysis, % DM)	5.7	5.8	5.5
Ash (% DM)	3.9	4.7	6.0
Crude fiber (% DM)	4.4	4.5	4.8
Neutral detergent fiber (% fiber)	16.7	16.0	16.3
Acid detergent fiber (% fiber)	5.3	5.5	5.9
Carbohydrates (% DM)	66.2	60.5	53.3
Starch	43.1	36.7	33.9
Glucose	0.4	0.3	0.2
Fructose	0.4	0.3	0.2
Sucrose	2.8	3.0	3.2
Lactose	1.3	1.3	1.3
Metabolizable energy (kcal/g)	3.5	3.5	3.4

**^#^** Micronutrients were similar between all diets.

**Table 2:** Statistical comparisons of predominant bacterial phyla, families, and genera in maternal ceca, milk, and offspring ceca of rat dams fed a 10% protein (LP) or 20% protein (HP) diet. *p*-values were generated using general linear models (GLM).

Phyla/Family	Genera	*p*-Value		
		Diet	Sample Type	Interaction
**Actinobacteria**		0.290	**<0.001**	0.41
*Micrococcaceae*	*Rothia*	0.380	**<0.001**	0.39
**Bacteroidetes**		0.500	**<0.001**	0.88
*Bacteroidaceae*	*Bacteroides*	0.480	**<0.001**	0.31
*Porphyromonadaceae*	*Parabacteroides*	0.630	**<0.001**	0.91
*Rikenellaceae*	*Rikenellaceae*	0.970	**<0.001**	0.2
*S24-7*	*S24-7*	0.980	**<0.001**	0.24
**Firmicutes**		**0.005**	**<0.001**	0.42
*Planococcaceae*	Planococcaceae *	0.160	**<0.001**	0.06
*Staphylococcaceae*	*Staphylococcus*	0.370	**0.002**	0.32
*Lactobacillaceae*	*Lactobacillus*	**0.014**	**<0.001**	0.59
*Streptococcaceae*	*Streptococcus*	0.052	**<0.001**	0.16
*Clostridiales*	*Clostridiales ^&^*	0.300	**<0.001**	**0.013**
*Lachnospiraceae*	Lachnospiraceae *	0.440	**<0.001**	0.08
*Ruminococcaceae*	Ruminococcaceae *	**0.001**	**<0.001**	0.07
*Ruminococcaceae*	*Oscillospira*	0.570	**<0.001**	0.2
*Ruminococcaceae*	*Ruminococcus*	**0.003**	**<0.001**	**0.026**
**Proteobacteria**		0.910	**<0.001**	0.97
*Rhizobiaceae*	*Agrobacterium*	1	**<0.001**	1
*Comamonadaceae*	*Delftia*	0.340	**<0.001**	0.26
*Enterobacteriaceae*	*Enterobacteriaceae*	0.600	0.001	0.85
*Pasteurellaceae*	*Other*	0.840	**<0.001**	0.93
*Pasteurellaceae*	*Haemophilus*	**0.044**	**<0.001**	0.062
*Pasteurellaceae*	*Mannheimia*	**0.005**	**<0.001**	**<0.001**
*Moraxellaceae*	*Acinetobacter*	0.740	0.068	0.43
*Xanthomonadaceae*	Xanthomonadaceae *	0.480	**<0.001**	0.48
**Verrucomicrobia**		**0.002**	**<0.001**	**<0.001**
*Verrucomicrobiaceae*	*Akkermansia*	**0.002**	**<0.001**	**<0.001**

* Unclassified OTU families, ^&^ Unclassified OTU order of *Clostridiales*. Values in bold indicate statistical difference (*p* < 0.05).

**Table 3:** Predominant bacterial phyla, families, and genera (percentage of reads) in maternal ceca, milk, and offspring ceca of rat dams fed a 10% protein (LP) or 20% protein (HP) diet.

Phyla/Family	Genera	Maternal Cecal		Milk		Offspring Cecal	
		LP	HP	LP	HP	LP	HP
**Actinobacteria**		1.4 ± 1.1%	0.1 ± 0.0%	26.0 ± 0.0%	19.2 ± 0.1%	1.3 ± 0.2%	1.0 ± 0.2%
*Micrococcaceae*	*Rothia*	neg	neg	24.8 ± 4.3%	18.2 ± 7.0%	1.1 ± 0.2%	0.9 ± 0.2%
**Bacteroidetes**		24.8 ± 3.5%	21.5 ± 3.6%	neg	neg	30.8 ± 3.0%	29.1 ± 2.7%
*Bacteroidaceae*	*Bacteroides*	4.2 ± 0.9%	7.2 ± 1.4%	neg	neg	16.1 ± 3.6%	11.7 ± 1.7%
*Porphyromonadaceae*	*Parabacteroides*	neg	neg	absent	absent	10.6 ± 2.0%	11.9 ± 1.8%
*Rikenellaceae*	*Rikenellaceae*	5.8 ± 1.1%	4.2 ± 1.2%	neg	neg	0.3 ± 0.1%	1.1 ± 0.7%
*S24-7*	*S24-7*	11.9 ± 2.2%	9.4 ± 1.7%	absent	absent	3.2 ± 0.5%	4.4 ± 1.0%
**Firmicutes**		66.1 ± 2.8%	71.9 ± 1.5%	33.9 ± 0.1%	39.7 ± 0.1%	39.2 ± 4.2%	54.9 ± 2.1%
*Planococcaceae*	Planococcaceae *	neg	neg	6.7 ± 3.4%	1.9 ± 0.6%	neg	neg
*Staphylococcaceae*	*Staphylococcus*	absent	absent	8.8 ± 5.5%	3.8 ± 1.7%	neg	neg
*Lactobacillaceae*	*Lactobacillus*	0.3 ± 0.0%	4.7 ± 3.0%	0.8 ± 0.1%	12.5 ± 5.1%	19.6 ± 2.6%	25.6 ± 3.8%
*Streptococcaceae*	*Streptococcus*	neg	neg	15.3 ± 4.8%	8.4 ± 2.3%	2.8 ± 0.4%	1.0 ± 0.1%
*Clostridiales*	*Clostridiales ^&^*	26.6 ± 3.3%	18.6 ± 3.2%	0.2 ± 0.0%	3.0 ± 1.9%	2.8 ± 0.7%	3.4 ± 0.9%
*Lachnospiraceae*	Lachnospiraceae *	9.3 ± 2.1%	6.8 ± 1.8%	0.1 ± 0.0%	1.7 ± 0.9%	1.5 ± 0.2%	3.2 ± 0.6%
*Ruminococcaceae*	Ruminococcaceae *	6.1 ± 1.2%	14.4 ± 2.6%	0.1 ± 0.0%	1.6 ± 0.8%	3.4 ± 0.6%	8.9 ± 1.3%
*Ruminococcaceae*	*Oscillospira*	10.5 ± 1.1%	7.4 ± 1.5%	neg	neg	neg	neg
*Ruminococcaceae*	*Ruminococcus*	3.5 ± 0.8%	6.2 ± 1.3%	neg	neg	neg	neg
**Proteobacteria**		3.3 ± 0.6%	5.3 ± 3.2%	39.5 ± 0.1%	38.9 ± 0.1%	13.1 ± 2.3%	13.1 ± 3.5%
*Rhizobiaceae*	*Agrobacterium*	absent	absent	18.8 ± 6.4%	18.9 ± 6.1%	absent	absent
*Comamonadaceae*	*Delftia*	absent	absent	1.0 ± 0.4%	1.6 ± 0.5%	absent	absent
*Enterobacteriaceae*	*Enterobacteriaceae*	neg	neg	neg	neg	6.5 ± 2.3%	8.6 ± 2.8%
*Pasteurellaceae*	*Other*	absent	absent	4.4 ± 1.1%	5.3 ± 3.9%	neg	neg
*Pasteurellaceae*	*Haemophilus*	neg	neg	0.8 ± 0.3%	2.2 ± 0.9%	neg	neg
*Pasteurellaceae*	*Mannheimia*	absent	neg	5.3 ± 1.3%	2.3 ± 0.6%	neg	neg
*Moraxellaceae*	*Acinetobacter*	0.3 ± 0.1%	3.6 ± 3.4%	neg	neg	4.4 ± 1.2%	3.7 ± 1.5%
*Xanthomonadaceae*	Xanthomonadaceae *	absent	absent	4.68 ± 1.32%	3.54 ± 0.98%	absent	absent
**Verrucomicrobia**		1.4 ± 0.8%	0.01 ± 0.0%	absent	absent	15.2 ± 3.1%	0.4 ± 0.2%
*Verrucomicrobiaceae*	*Akkermansia*	1.4 ± 0.8%	0.01 ± 0.0%	absent	absent	15.2 ± 3.1%	0.4 ± 0.2%

* Unclassified OTU families, ^&^ Unclassified OTU order of *Clostridiales*, neg = negligible (<1% abundance), Absent = not detected.

## Supplementary Materials

Raw sequence data from preliminary study and main study are deposited in https://datadryad.org/ and available online. The following are available online at https://www.mdpi.com/2072-6643/11/9/2257/s1, Figure S1: Non-metric multidimensional scaling (nMDS) plot of rat (**a**) dam cecum and (**b**) pup cecum grouped by diet. The 10% protein (LP) diet samples denoted by blue triangles and 20% protein (HP) diet samples denoted by red circles. Bacterial OTUs were clustered using Bray–Curtis. Stress was (**a**) 9.16 × 10^−5^ and (**b**) 0.17, Figure S2: Rarefaction curve of unique OTUs for the milk and cecum of rats consuming a 10% protein (LP, blue shapes) vs. 20% protein (HP, orange shapes) diet, and their pups. Lines with triangles denote dam ceca, lines with circles denote pup ceca, and lines with squares denote dam milk, Figure S3: Comparison of bacterial OTUs of the feces of rats consuming a 10% protein (LP, blue bars/shapes), 15% protein (MP, green bars/shapes), and a 20% protein (HP, orange bars/shapes) diet. (**a**) Rarefaction curve of unique OTUs is given by diet. A broken line with triangle denotes LP (overlapped by MP), dotted line with square denotes MP, and line with circle denotes HP. The plot shows means and standard error bars. (**b**) Alpha diversity by diet using the Shannon–Wiener Index. (**c**) Alpha diversity by diet using phylogenetic distance. (**d**) Alpha diversity by diet using unique OTUs. Bar graphs show means and standard error bars. Asterisk indicates statistical difference (GLM, * *p* < 0.05), Figure S4: Non-metric multidimensional scaling (nMDS) plot of rat fecal samples between diets and time. The 10% protein (LP) diet samples denoted by green shapes, 15% protein (MP) diet samples denoted by blue shapes, and 20% protein (HP) diet samples denoted by red shapes. Bacterial OTUs were clustered using Bray–Curtis. Stress was 0.14, Table S1: Predominant bacterial phyla and genera (percentage of readings) in rat feces fed a low-protein (LP), moderate-protein (MP), or high-protein (HP) diet. Mean values with their standard errors are given. *p*-values were generated using Kruskal–Wallis ranked-sum test, and Monte–Carlo permutations were done 999 times. Bonferroni correction (BF) *p*-values were also generated. Values in bold indicate statistical difference (*p* < 0.05).

## Appendix A. Fecal Microbiota Experiment

### Appendix A.1. Methods

Twelve seven-week-old female rats were randomly assigned to each of three dietary treatment groups, low-protein (LP), moderate-protein (MP), and high-protein (HP) (*n* = 4 rats/diet). After one week on the experimental diets, fecal pellets were collected every three days from each rat. If a rat did not produce any feces at the time of collection, then the collection was attempted again within 24 h. For sample collection, a sterilized piece of foil was placed under each rat, and the rat was stimulated to defecate onto the foil by gently pressing the formed pellets towards the anus. Fecal pellets were placed in sterile microcentrifuge tubes using sterile forceps and then were placed in an −80 °C freezer for future DNA extractions. The extraction of fecal DNA followed the same method as described from cecal DNA in the method section. Similarly, the pipeline for bioinformatics for fecal samples was conducted the same way as for cecal and milk samples.

Comparisons of fecal bacterial phyla and genera relative abundances between treatment groups were completed using Kruskal–Wallis test because most OTUs were not normally distributed; Monte–Carlo permutations for all Kruskal–Wallis tests were done 999 times. General linear models (GLM) were used to compare diet and time effects between samples. Analysis of similarities (ANOSIM) was used to compare if microbial communities were dissimilar between treatment groups; identical communities were given an *R* statistic close to 0, and dissimilar communities were given a value close to 1. Bray–Curtis was used to quantitatively determine differences in fecal communities and for non-metric multidimensional scaling (nMDS). Statistical significance was established when *p* < 0.05.

### Appendix A.2. Results

A total of 2,598,881 reads were produced from 46 fecal samples by 16S rRNA gene amplicon sequencing rat fecal DNA after quality-filtering and a mean sample depth of 56,497 reads with a standard deviation of 7,970 reads from fecal samples. A total of 44,540 randomly selected sequences were evaluated from all samples to provide estimates for alpha and beta diversity. Rarefaction plot for unique OTUs reached an asymptote, indicating the selected sampling depth would be sufficient to include most or all OTUs for all samples (Figure S3a). Alpha diversity (i.e., phylogenetic distance, F = 6.50, df = 2, 43, *p* = 0.0034), Shannon–Wiener Index (F = 3.61, df = 2, 43, *p* = 0.035), and unique OTUs of bacteria (F = 7.01, df = 2, 43, *p* = 0.002) in feces of rats varied with diet (Figure S3b–d). Feces of rats consuming LP diet displayed highest mean alpha diversity and unique OTUs, but these values were not statistically different from rats consuming MP diet. Feces of rats consuming HP diet exhibited a lower alpha diversity and unique OTUs relative to the other two groups. No temporal variation was observed within treatment groups in the alpha diversity of fecal samples (Shannon: F = 0.17, df = 5, 40, *p* = 0.92; Phylogenetic distance: F = 0.69, df = 5, 40, *p* = 0.56; Unique OTUs: F = 0.42, df = 5, 40, *p* = 0.42).

Diet had an effect on bacterial communities in rat feces when measured by Bray–Curtis (*p* = 0.001; *r* = 0.66). nMDS plot had the stress of 0.14, and while clusters were overlapping, LP and HP samples had separation with MP samples in between both of them. This analysis suggested the fecal bacterial community for rats consuming the MP diet was not different from the other two groups (Figure S4). OTU relative abundance differences between diets were largely driven by Bacteroidetes (particularly *Rikenellaceae* and *S24-7*), which decreased in relative abundance as dietary protein increased. Actinobacteria was also higher in feces of LP rats, but the contribution of this phylum was small relative to other identified phyla. Percent of microorganisms belonging to phylum Firmicutes increased as dietary protein intake with *Clostridales* and *Ruminococcaceae* plays the most prominent role in this pattern (Table S1).
