# Supplementary material for: Maternal Dietary Protein Intake Influences Milk and Offspring Gut Microbial Diversity in a Rat (Rattus norvegicus) Model
Source: Nutrients. 2019 Sep 19;11(9):2257. doi: 10.3390/nu11092257 (PMC6769776; doi:10.3390/nu11092257)
Supplement: Supplementary file 1 [file nutrients-11-02257-s001.pdf]

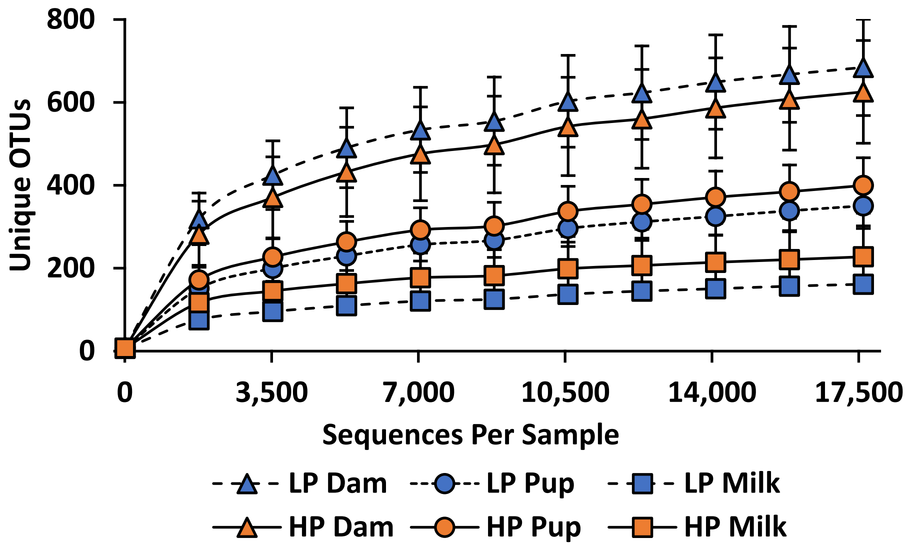

Figure S1. Rarefaction curve of unique OTUs for the milk and cecum of rats consuming a 10% protein (LP, blue shapes) vs 20% protein (HP, orange shapes) diet, and their pups. Lines with triangles denotes dam ceca, lines with circles denote pup ceca, and lines with squares denote dam milk.

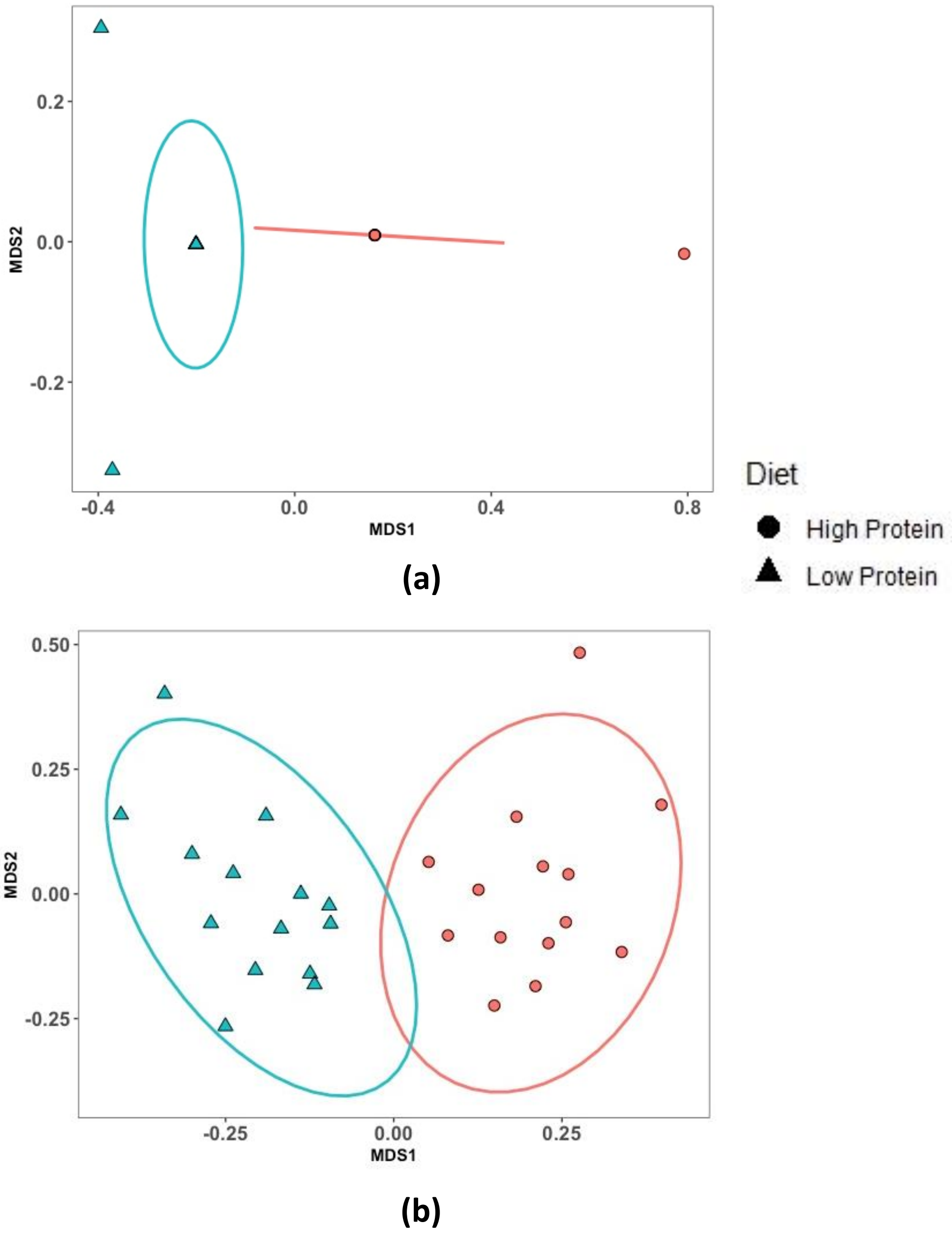

Figure S2. Non-metric multidimensional scaling (nMDS) plot of rat **(a)** dam cecum and **(b)** pup cecum grouped by diet. 10% protein (LP) diet samples denoted by blue triangles and 20% protein (HP) diet samples denoted by red circles. Bacterial OTUs were clustered using Bray-Curtis. Stress was **(a)** $9.16x10^{-5}$ and **(b)** 0.17.

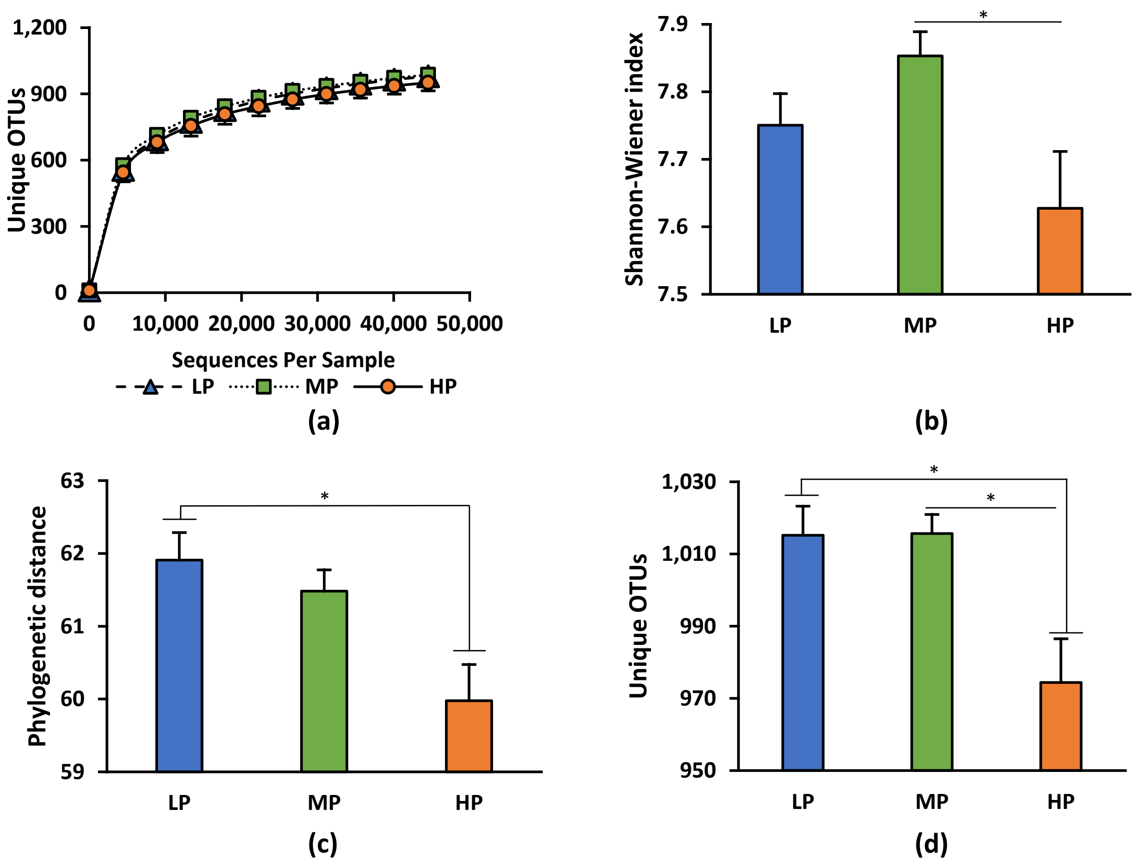

Figure S3. Comparison of bacterial OTUs of the feces of rats consuming a 10% protein (LP, blue bars/shapes), 15% protein (MP, green bars/shapes), and a 20% protein (HP, orange bars/shapes) diet. **(a)** Rarefaction curve of unique OTUs are given by diet. Broken line with triangle denotes LP (overlapped by MP), dotted line with square denotes MP, and line with circle denotes HP. Plot shows means and standard error bars. (**b**) Alpha diversity by diet using Shannon-Wiener Index. (**c**) Alpha diversity by diet using phylogenetic distance. (**d**) Alpha diversity by diet using unique OTUs. Bar graphs show means and standard error bars. Asterisk indicates statistical difference (GLM, * $p < 0.05$).

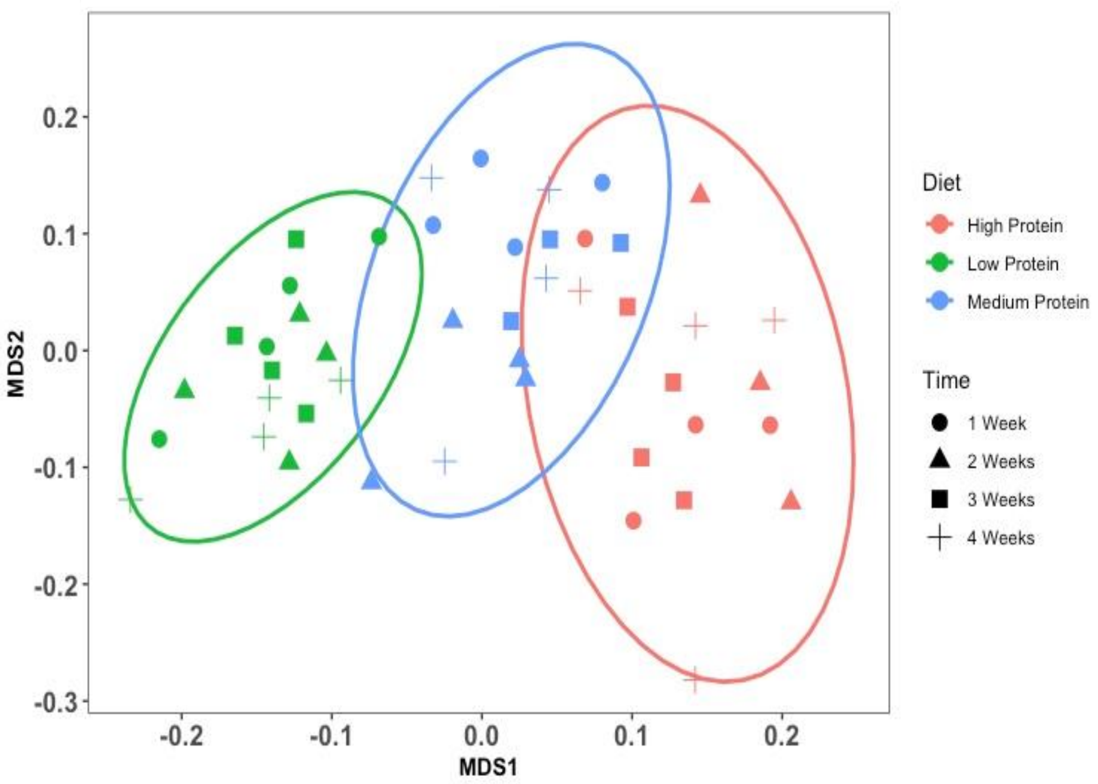

Figure S4. Non-metric multidimensional scaling (nMDS) plot of rat fecal samples between diets and time. 10% protein (LP) diet samples denoted by green shapes, 15% protein (MP) diet samples denoted by blue shapes, and 20% protein (HP) diet samples denoted by red shapes. Bacterial OTUs were clustered using Bray-Curtis. Stress was 0.14.

Table S1. Predominant^ bacterial phyla and genera (percentage of readings) in rat feces fed a low-protein (LP), moderate-protein (MP), or high-protein (HP) diet. Mean values with their standard errors are given. P-values were generated using Kruskal-Wallis ranked-sum test and Monte-Carlo permutations were done 999 times. Bonferroni correction (BF) p-values were also generated. Bolded numerical values indicate statistical difference (P < 0.05).

**Feces**			**Diet**		X^2	**P-Value**	**BF**
**Phyla/*Family***	**Genera**	**LP**	**MP**	**HP**			**P-Value**
**Actinobacteria**		9.5%	10.4%	13.7%	30.0	**<0.0001**	**0.001**
Bifidobacteriaceae	*Bifidobacterium*	1.6%	0.0%	0.0%	31.4	**<0.0001**	**0.001**
Bacteroidetes		9.5%	10.4%	13.7%	14.2	**0.0008**	**0.01**
Bacteroidaceae	*Bacteroides*	5.8%	5.3%	4.8%	1.7	0.4	1
Rikenellaceae	*Rikenellaceae*	5.9%	5.1%	3.2%	19.3	**<0.0001**	**0.001**
S24-7	*S24-7*	17.8%	12.2%	12.8%	16.2	**0.0003**	**0.004**
**Firmicutes**		9.5%	10.4%	13.7%	17.8	**0.0001**	**0.001**
Lactobacillaceae	*Lactobacillus*	2.7%	1.9%	3.2%	3.9	0.1	1
Clostridiales	*Clostridiales*[&]	25.5%	35.2%	30.5%	17.3	**0.0002**	**0.003**
	Clostridiaceae	1.5%	0.1%	0.1%	29.9	**<0.0001**	**0.001**
Lachnospiraceae	*Other*	0.3%	1.4%	1.5%	21.5	**<0.0001**	**0.001**
Lachnospiraceae	*Lachnospiraceae**	4.4%	4.2%	4.7%	1.1	0.6	1
Ruminococcaceae	*Ruminococcaceae**	9.5%	10.4%	13.7%	6.8	**0.03**	0.4

^Only includes those OTUs where at least one diet displayed a relative abundance of > 0.50%. *Unclassified OTU families, [&]Unclassified OTU order of *Clostridiales*
